# Intramolecular Energy and Solvent‐Dependent Chirality Transfer within a BINOL‐Perylene Hetero‐Cyclophane

**DOI:** 10.1002/anie.202206706

**Published:** 2022-06-21

**Authors:** Guanghui Ouyang, Jessica Rühe, Yang Zhang, Mei‐Jin Lin, Minghua Liu, Frank Würthner

**Affiliations:** ^1^ Universität Würzburg Institut für Organische Chemie & Center for Nanosystems Chemistry Am Hubland 97074 Würzburg Germany; ^2^ CAS Key Laboratory of Colloid Interface and Chemical Thermodynamics Institute of Chemistry Chinese Academy of Sciences ZhongGuanCun, North First Street 2 100190 Beijing China; ^3^ State Key Laboratory of Photocatalysis on Energy and Environment College of Chemistry Fuzhou University 350116 Fuzhou China

**Keywords:** Chirality Transfer, Cyclophanes, Energy Transfer, Perylene Bisimide, Solvent Effects

## Abstract

Multichromophoric macrocycles and cyclophanes are important supramolecular architectures for the elucidation of interchromophoric interactions originating from precise spatial organization. Herein, by combining an axially chiral binaphthol bisimide (BBI) and a bay‐substituted conformationally labile twisted perylene bisimide (PBI) within a cyclophane of well‐defined geometry, we report a chiral PBI hetero‐cyclophane (**BBI**‐**PBI**) that shows intramolecular energy and solvent‐regulated chirality transfer from the BBI to the PBI subunit. Excellent spectral overlap and spatial arrangement of BBI and PBI lead to efficient excitation energy transfer and subsequent PBI emission with high quantum yield (80–98 %) in various solvents. In contrast, chirality transfer is strongly dependent on the respective solvent as revealed by circular dichroism (CD) spectroscopy. The combination of energy and chirality transfer affords a bright red circularly polarized luminescence (CPL) from the PBI chromophore by excitation of BBI.

## Graphical abstract

A chiral perylene bisimide (PBI) hetero‐cyclophane comprising a chiral binaphthol bisimide (BBI) dye and a dynamically racemic PBI dye shows quantitative energy and solvent‐dependent chirality transfer among its components, thereby leading to an amplified bright red circularly polarized luminescence from PBI.

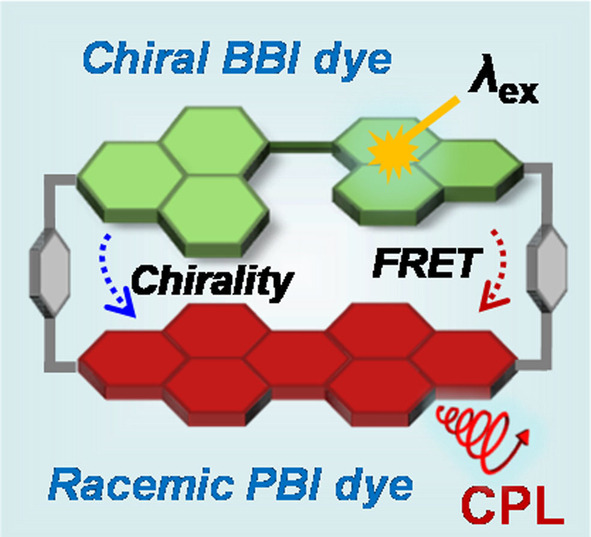

Multichromophoric supramolecular systems^[1]^ have attracted a lot of interest across the chemical community because they offer advantageous properties compared to single chromophores such as improved panchromatic absorption of solar light,^[2]^ excitation energy transfer^[3]^ or new photophysical pathways such as charge separation or singlet fission.^[4]^ Precise arrangement of heterogeneous chromophores in such supramolecular systems is the key issue to control the interchromophoric interactions and accordingly improve the performances for the respective application.^[5]^ In this context, hetero‐cyclophanes are ideal scaffolds because of their well‐defined molecular geometry and precise spatial organization of chromophores.^[6]^ Furthermore, a π‐cavity provided by hetero‐cyclophanes might bind guests or accommodate solvents,^[7]^ thereby allowing control over interchromophoric interactions. However, only few reports are available on guest‐ or solvent‐regulated interchromophoric interactions and concomitant photophysical processes in (hetero‐)cyclophanes.[^8^, ^9^]

As outstanding luminophores with high quantum yield, excellent (photo‐)stability and large π‐surface, perylene bisimides (PBIs)^[10]^ have been utilized in functional homo‐ and hetero‐cyclophanes for multi‐purpose applications.^[11]^ Furthermore, at bay positions substituted PBIs are chiral but prevail as conformationally labile mixtures of *M*‐ and *P*‐PBI atropisomers due to low activation barriers for core twisting of around 60 kJ mol^−1^.[^12^, ^13^] Previous experiments from our laboratory indicated that CD signals of PBI chromophore in an achiral PBI (homo‐)cyclophane could be induced via chirality transfer from chiral guests.^[14]^ Encouraged by this work we conjectured that combining a chiral luminophore with at bay positions with four phenoxy substituents functionalized PBI dye should provide a promising molecular scaffold to study solvent‐dependent interchromophoric interactions, in particular energy and chirality transfer.^[15]^ Here, we report the first chiral PBI hetero‐cyclophane consisting of a recently developed chiral 1,1′‐bi(2‐naphthol‐4,5‐dicarboximide) (BBI) luminophore^[16]^ and a racemic PBI dye, which shows quantitative Förster resonance energy transfer (FRET) and solvent‐regulated chirality transfer among its components (Figure 1).^[17]^

**Figure 1:**
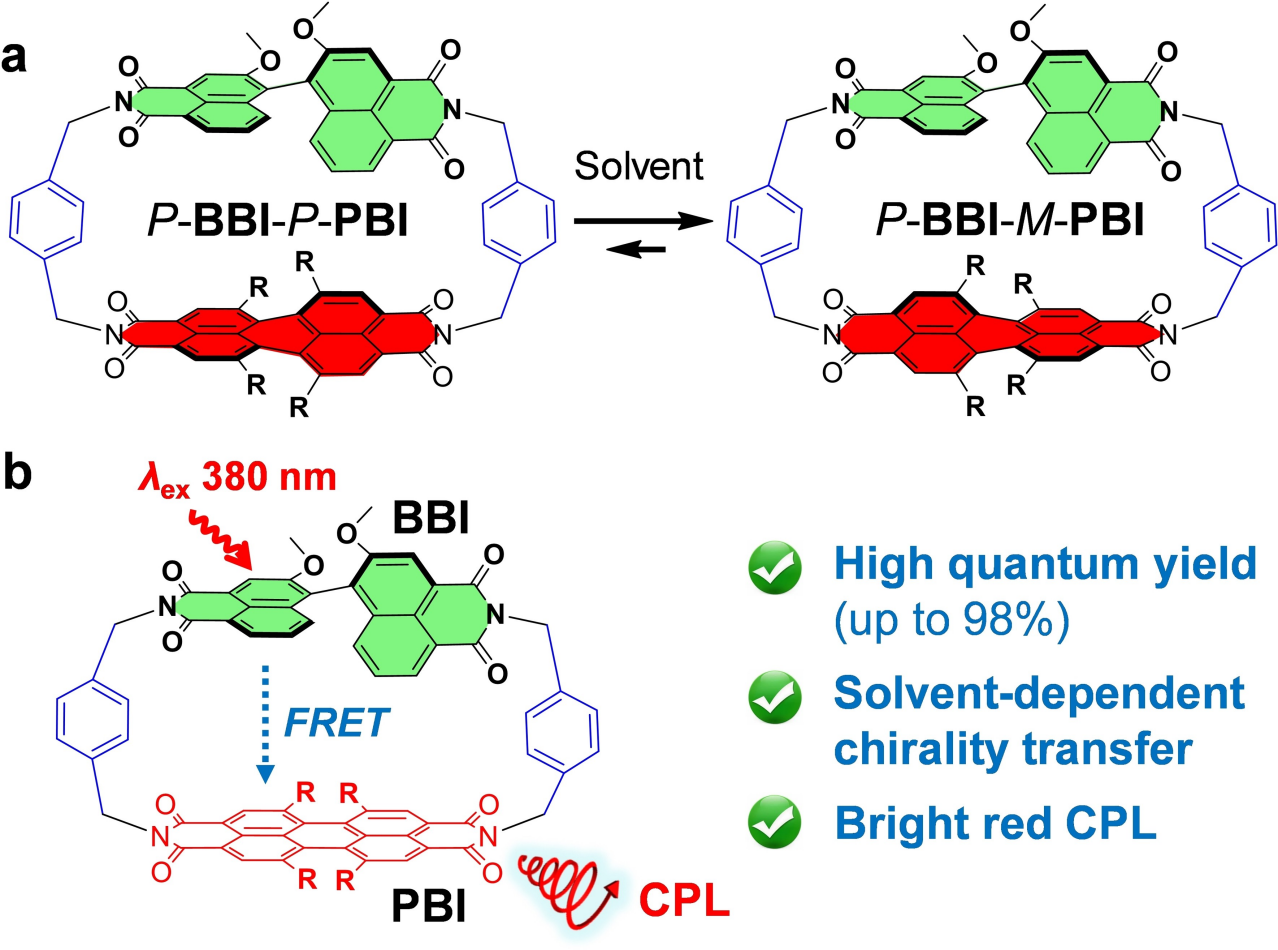
Schematic illustration of a) chirality transfer and b) excitation energy transfer from the chiral *P*‐BBI^[17]^ unit to bay‐substituted twisted PBI chromophores within cyclophane. R=4‐*tert*‐butylphenoxy.

The chiral **BBI**‐**PBI** hetero‐cyclophane is synthesized as shown in Scheme 1a. Imidization of PBI **1** with Boc‐protected *p*‐xylylenediamine **2**, followed by deprotection affords intermediate **3** in 95 % yield. Subsequent macrocyclization with racemic BBI **4** is less satisfying with a yield of only 8 % after purification by column and recycling gel permeation chromatography (for details see Supporting Information). Resolution on a semi‐preparative chiral column affords two enantiomers *M*‐**BBI**‐**PBI** and *P*‐**BBI**‐**PBI** for which the PBI chirality is under the influence of the BBI unit due to the low interconversion barrier for PBI atropisomers (Figure S2 and S3).

**Scheme 1:**
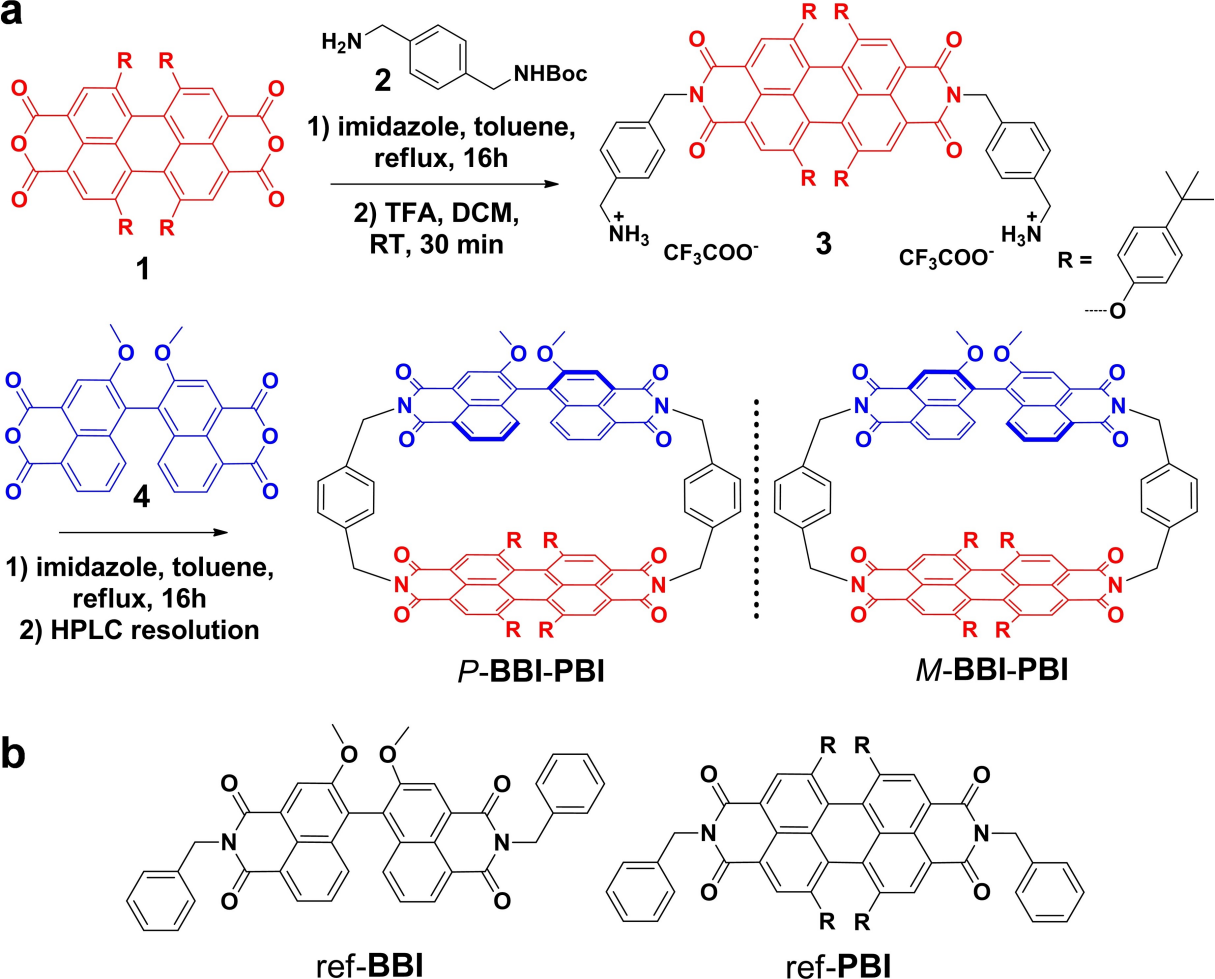
a) Synthetic route to **BBI**‐**PBI** cyclophane. b) Chemical structures of two reference compounds, ref‐**BBI** and ref‐**PBI**, R=4‐*tert*‐butylphenoxy.

As demonstrated for the two reference compounds (Scheme 1b), both BBI and PBI are outstanding emitters with fluorescence quantum yields of 68 % and 91 % in CH_2_Cl_2_, respectively (Table S1). Due to a perfect overlap of BBI emission and PBI absorption bands (Figure 2a, S4a), upon excitation of the BBI subunit of **BBI‐PBI** cyclophane at 380 nm an almost quantitative FRET^[18]^ is observed, affording exclusive emission from the PBI subunit (Figure 2b, S5). The high efficiency of this FRET is also corroborated by the excitation spectrum (Figure 2b, red line) that demonstrates that PBI emission is equally intense irrespective of the excitation wavelength, i.e. absorption by BBI or PBI subunit, as well as our calculations (Figure S6). Fluorescence quantum yields (FLQYs) and lifetimes of **BBI**‐**PBI** remain within the range of 80–98 % and 6.9–7.7 ns for the investigated solvents from nonpolar *p*‐xylene to polar acetonitrile (Figure S7 and Table S2), indicating that there is little influence of the solvent on the FRET process.

**Figure 2:**
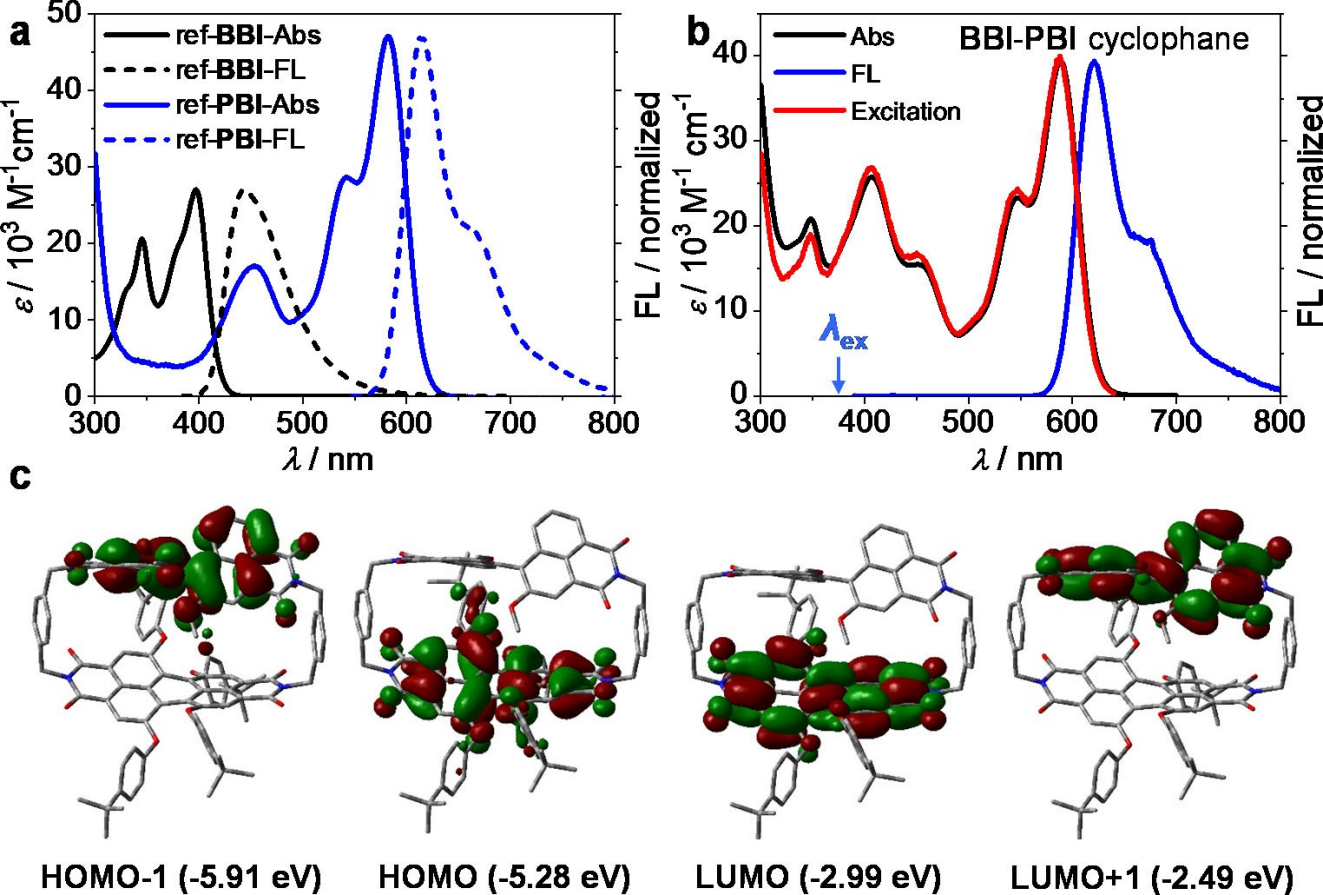
Optical spectra and frontier molecular orbitals. a) UV/Vis and FL spectra of ref‐**BBI** (black lines, for FL, *λ*_ex_=380 nm) and ref‐**PBI** (blue lines, for FL, *λ*_ex_=550 nm) in CH_2_Cl_2_, 293 K. For UV/Vis, [ref‐**BBI**]=[ref‐**PBI**]=3.4×10^−5^ M. b) UV/Vis (black line), FL (blue line, *λ*_ex_=380 nm) and excitation spectra (red line, *λ*_em_=650 nm) of **BBI**‐**PBI**. For UV/Vis [**BBI**‐**PBI**]=1.8×10^−5^ M. c) Energy levels and molecular orbitals of *P*‐**BBI**‐*M*‐**PBI** cyclophane calculated by Gaussian 16 at B3LYP 6‐311G (d,p) level.^[20]^

Density functional theory (DFT) calculations show that both HOMO and LUMO orbitals of **BBI**‐**PBI** cyclophane are localized on the PBI moiety, while LUMO+1 and HOMO‐1 are localized on the BBI subcomponent (Figure 2c). Despite of the close face‐to‐face orientation, all molecular orbitals are well separated and located on either the BBI or the PBI subunits and with almost equal energy levels than those calculated for their reference counterparts (Figure S4b, S8, S9 and Table S3). For this situation charge transfer processes[^11e^, ^19^] do not play a role and intense fluorescence persists even in polar solvents.

The chiroptical properties of **BBI**‐**PBI** cyclophane were studied by both CD and CPL spectroscopies to evaluate intramolecular chirality transfer from BBI to PBI. For all investigated solvents the two HPLC fractions of **BBI**‐**PBI** cyclophane (Figure S3) give typical mirror‐image Cotton effects (Figure 3a, b, S10, S11) that clearly demonstrate that the two HPLC peaks correspond to a pair of enantiomers (blue and red lines, the first and second fractions, respectively). However, a pronounced solvent dependency is observed for the chirality transfer efficiency from axially chiral BBI to the conformationally labile PBI as exemplified in Figure 3a, b for CHCl_3_ and CH_2_Cl_2_. In CHCl_3_ (as well as CHBr_3_, Figure S10b) the PBI chromophore does not show any CD signal between 500 and 650 mm where this PBI dye has its main S_0_→S_1_ absorption band, indicating an unbiased equilibrium between *P*‐**PBI** and *M*‐**PBI** atropisomers. Therefore, the Cotton effects of **BBI**‐**PBI** cyclophane in CHCl_3_ should be exclusively attributed to the chiral BBI chromophores. Based on our previous CD spectroscopic analyses for enantiopure BBI,^[16]^ the red and blue curves in Figure 3a correspond to *P*‐**BBI**‐**PBI** and *M*‐**BBI**‐**PBI**, respectively. In contrast, while the BBI Cotton effects are preserved, the PBI chromophore shows induced CD signals for the S_0_→S_1_ transition in CH_2_Cl_2_ and most other investigated solvents including various chlorinated ethanes, CCl_4_, toluene, *p*‐xylene and THF with absorption dissymmetry factors |g_PBI_|=Δ*ϵ*/*ϵ* in the range of 1.2×10^−4^ to 2.3×10^−4^ (Figure S11, Table 1). These monosignate Cotton effects for the PBI S_0_→S_1_ transition allow us to assign the helical screw sense of the twisted PBIs and to estimate the degree of chirality transfer based on our earlier work for an enantiopure *P*‐helical tetraphenoxy‐substituted PBI with a positive Cotton effect of Δ*ϵ*=+65 M^−1^cm^−1^ and an absorption dissymmetry factor g_abs_=0.0018.^[13]^ Accordingly, the opposite helical screw sense is preferably induced in *P*‐**BBI‐PBI** from *P*‐BBI to the neighboring PBI subunit with an enantiomeric excess (ee) of up to around 0.12 in CH_2_Cl_2_. Decreasing the temperature to 263 K could enhance the ee value to about 0.18 in CH_2_Cl_2_ (Figure S12). Time‐dependent DFT computation results of *P*‐**BBI**‐*M*‐**PBI** isomer (Figure S13) afford a negative Cotton effect at the S_0_→S_1_ transition of PBI (centered near 600 nm), which is in agreement with our helicity assignment based on experimental results (Figure 3b, red line).

**Figure 3:**
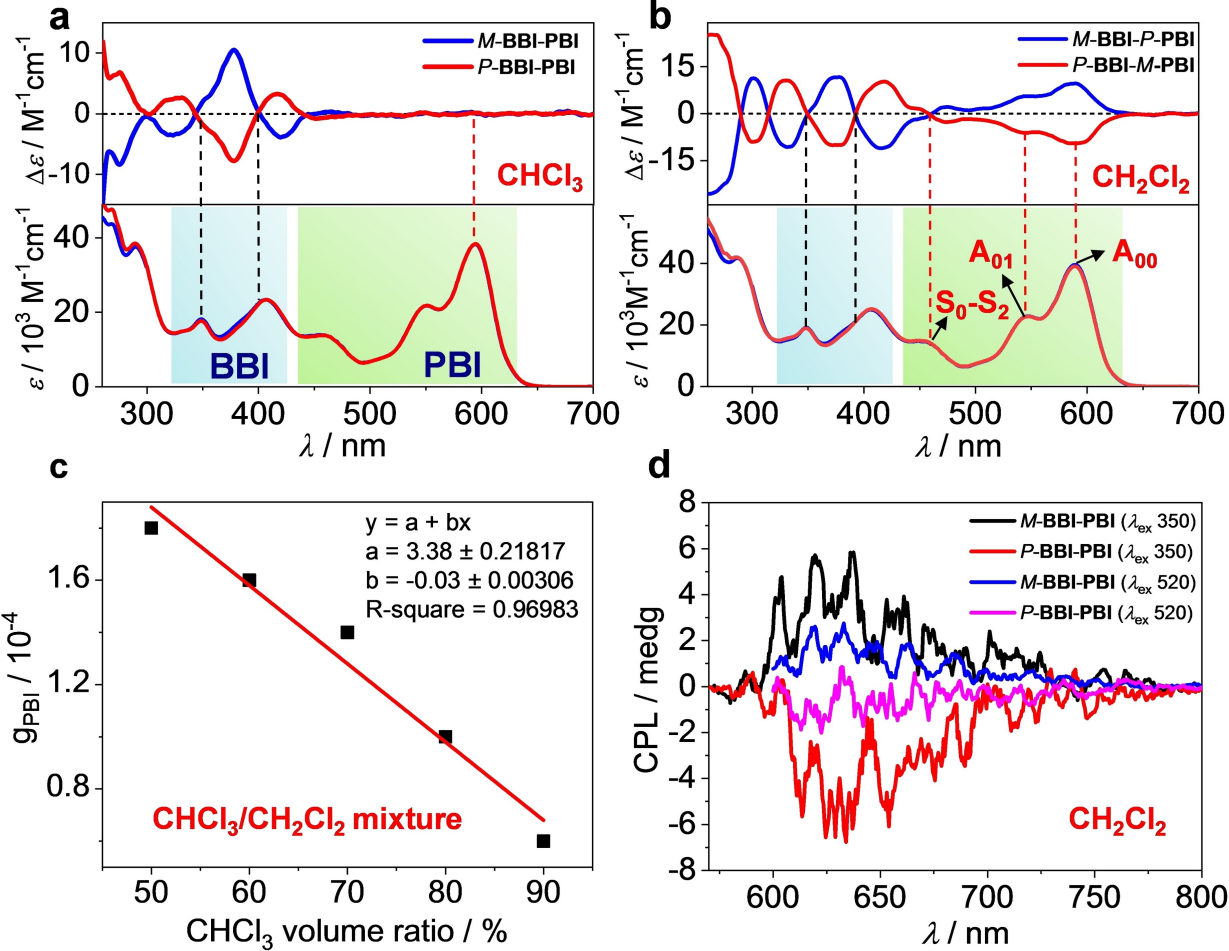
Chiroptical spectra to study solvent‐dependent chirality transfer. CD spectra of **BBI**‐**PBI** enantiomers in a) CHCl_3_, and b) CH_2_Cl_2_. A_00_, A_01_ vibronic transitions for the S_0_–S_1_ and S_0_–S_2_ transition range for PBI chromophore shaded in green color and absorption bands mainly attributable to BBI shaded in blue color. c) Plots of g_PBI_ of *P*‐**BBI**‐**PBI** against CHCl_3_ volume ratio in CHCl_3_/CH_2_Cl_2_ mixture, [*P*‐**BBI**‐**PBI**]=1.7×10^−5^ M, cuvette path length 10 mm, 293 K. d) CPL spectra of **BBI**‐**PBI** enantiomers in CH_2_Cl_2_ by excitation at 350 nm or 520 nm. [**BBI**‐**PBI**]=2.3×10^−4^ M, cuvette path length 1 mm, 293 K.

**Table 1:** Chiroptical data of *P*‐**BBI**‐**PBI** cyclophane in different solvents.

Solvents	g_BBI_^[a]^ [×10^−4^]	Δ*ϵ*‐PBI [M^−1^ cm^−1^]	g_PBI_^[b]^ [×10^−4^]	|g_PBI_|/ |g_PBI‐chiral_|^[c]^
CHCl_3_	8	0	0	0
CHBr_3_	8.5	0	0	0
CH_2_ClCHCl_2_	8.6	−4.9	−1.2	0.07
CH_2_ClCH_2_Cl	8.6	−5.6	−1.3	0.07
CHCl_2_CHCl_2_	6.4	−6.4	−1.4	0.08
CCl_4_	6.6	−5.8	−1.4	0.08
toluene	8.6	−7.8	−2	0.11
THF	8.4	−8.5	−2.1	0.12
CH_2_Cl_2_	8	−9.4	−2.1	0.12
*p*‐xylene	7.4	−8.6	−2.3	0.13
*R*‐limonene	n.d.^[d]^	0	0	0
*S*‐limonene	n.d.^[d]^	−23.2	−6.2	0.34

[a], [b] g_BBI_ and g_PBI_ represent the absorption dissymmetry factors of BBI and PBI chromophores, respectively. [c] The |g_PBI_|/|g_PBI‐chiral_| ratio corresponds to the optical purity, i.e. enantiomeric excess. Based on our earlier work^[13]^ we assume a value of g_PBI‐chiral_=0.0018 for an enantiopure *P*‐helical tetraphenoxy‐substituted reference PBI. [d] Not determinable because of overlap with CD signal of limonene.

To understand the significant solvent effects in controlling intramolecular chirality transfer within the cyclophane, control experiments by adding the chirality “quenching” solvent CHCl_3_, into CH_2_Cl_2_ are conducted. Increasing the volume ratio of CHCl_3_ in CHCl_3_/CH_2_Cl_2_ mixtures indeed leads to a linear decrease of the absorption dissymmetry factor for the PBI subunit for *P*‐**BBI**‐**PBI** cyclophane (Figure 3c and S14). Accordingly, the respective solvents obviously interact with the cleft‐like molecular structure of *P*‐**BBI**‐**PBI** cyclophane, thereby inducing a different bias on the chirality transfer as quantified by the |g_PBI_|/|g_PBI‐chiral_| ratio in Table 1. Chiral macrocycles are expected to have selective recognition toward chiral guests or solvents. The significant solvent‐regulated intramolecular chirality transfer of BBI‐PBI cyclophane encourages us to also investigate the influence of chiral solvents. Here we have found that the chirality transfer process from BBI to PBI could be affected by chirality match of the cyclophane and chiral solvents. Thus, *P*‐**BBI**‐**PBI** is preferably present as *P*‐**BBI**‐*M*‐**PBI** isomer in *S*‐limonene (Figure S15a, green line), giving a negative induced CD signal at PBI S_0_→S_1_ absorption band with a significantly increased enantiomeric excess value compared to the achiral solvents studied before (0.34, Table 1). However, no chirality transfer is observed from BBI to PBI in *R*‐limonene (Figure S15a, orange line). As expected, *M*‐**BBI**‐**PBI** only shows successful chirality transfer in *R*‐limonene (Figure S15b, orange line), giving mirror‐image Cotton effects to those in *P*‐**BBI**‐**PBI**/*S*‐limonene system. These results may be interpreted in the framework of Fischer's lock‐and‐key concept, i.e. that structural complementarity between the cyclophane solute and the respective solvent is needed to provide a sufficient impetus on the prevailing equilibrium between the two diastereomeric cyclophane conformations. As shown in this study the chirality transfer from BBI to PBI in **BBI**‐**PBI** cyclophane can be supported by both chiral and achiral solvents and appears to depend on the microscopic solvent‐solute interaction and non‐covalent supramolecular interactions within the cleft‐like cavity provided between BBI and PBI subunits (Figure S2).

The successful energy and chirality transfer from BBI to PBI combined with the intense PBI fluorescence emission open up the possibility for another unique experiment. Thus, to combine all of these features in one experiment, circularly polarized luminescence spectra are recorded. In CH_2_Cl_2_, photoexcitation of **BBI**‐**PBI** at *λ*_ex_=520 nm affords the expected red CPL signal at 630 nm with a luminescent dissymmetry factor *g*_lum_ of 3.5×10^−4^ (Figure 3c, blue and magenta lines). In contrast, photoexcitation of the energy donor BBI (*λ*_ex_=350 nm) affords a three times amplified bright CPL signal (*g*_lum_=9.6×10^−4^, Figure 3c, black and red lines; for CPL spectra in THF, see Figure S16a). While in CHCl_3_, due to a lack of chirality transfer from BBI to PBI no obvious CPL signal can be observed (Figure S16b). Therefore, the chirality transfer revealed by CPL measurements is in accordance with the CD data. The reason for the observed amplification of the CPL signal upon BBI excitation is at this stage not entirely clear but obviously relates to the fact that the absorbing BBI subunit is enantiopure whilst the emitting PBI subunit has an *ee* of only around 10 %. Similar energy transfer initiated CPL amplifications have recently been observed in self‐assembled chiral architectures.^[21]^

In summary, a new type of chiral luminescent hetero‐cyclophane comprising a chiral BINOL fluorophore and a core‐twisted PBI fluorophore has been introduced, wherein BBI acts as both energy donor and provider of chirality information. By means of sterical effects, the chiral BBI influences the conformational interconversion of *P*‐ and *M*‐PBI atropo‐enantiomers mediated by solvent molecules. In combination with the almost quantitative energy transfer from BBI to PBI due to excellent spectral overlap an amplification of circularly polarized luminescence could be observed from the PBI upon excitation of the BBI subunit. Accordingly, this work brought fundamental insights into dye–dye interactions which we believe might advance the prospects for chiral luminescent macrocycles in multiple‐channel information transfer and sensing applications.^[22]^

## Conflict of interest

The authors declare no conflict of interest.

## Supporting information

As a service to our authors and readers, this journal provides supporting information supplied by the authors. Such materials are peer reviewed and may be re‐organized for online delivery, but are not copy‐edited or typeset. Technical support issues arising from supporting information (other than missing files) should be addressed to the authors.

Supporting Information

## Data Availability

The data that support the findings of this study are available in the Supporting Information of this article.
