# Supplementary material for: Intramolecular Energy and Solvent‐Dependent Chirality Transfer within a BINOL‐Perylene Hetero‐Cyclophane
Source: Angew Chem Int Ed Engl. 2022 Jun 21;61(31):e202206706. doi: 10.1002/anie.202206706 (PMC9400993; doi:10.1002/anie.202206706)
Supplement: Supplementary file 1 — Supporting Information [file ANIE-61-0-s001.pdf]

Angewandte Chemie
Eine Zeitschrift der Gesellschaft Deutscher Chemiker

Supporting Information

Intramolecular Energy and Solvent-Dependent Chirality Transfer within a BINOL-Perylene Hetero-Cyclophane

*G. Ouyang, J. Rühe, Y. Zhang, M.-J. Lin, M. Liu, F. Würthner**

Supplementary Information

Contents

S1. General information 2

S2. Synthesis and characterization 2

S3. Supplementary figures 4

S4. Supplementary spectra 17

S5. References 19

S1. General information

Unless otherwise noted, all the chemical compounds were purchased from commercial suppliers and were directly used without further purification. Column chromatography was performed on silica gel (particle size 0.040−0.063 mm). NMR spectra were recorded on Bruker Avance III HD 400 MHz and 600 MHz spectrometers. NMR data analysis are presented as following, s: singlet, d: doublet, t: triplet, m: multiplet, br: broad signal. Recycling gel permeation chromatography (GPC) was carried out on a Shimadzu semi-preparative recycling chromatography setup with two or three semipreparative columns (Japan Analytical Industries Co., Ltd.; JAIGEL-2H and JAIGEL-2.5 H) with $CHCl_3$ as eluent. Recycling semipreparative HPLC was carried out on a JAI LC-9105 instrument. Chiral resolution was carried out using a Trentec Reprosil-100 Chiral-NR 8μm-column. Mass spectra were measured on a Bruker Daltonics micrOTOF-QIII focus instrument for high-resolution ESI or a Bruker Daltonics ultrafleXtreme mass spectrometer using DCTB as a matrix for high-resolution MALDI. UV/Vis absorption spectra were recorded on JASCO V-670 and V-770 spectrometers. Fluorescence spectra and lifetime measurements were measured with an Edinburgh Instruments FLS980 spectrometer. Lifetimes were measured using EPL picosecond pulsed diode laser (505.8 nm and 378.2 nm) as a light source. Fluorescence quantum yields were determined relative to common fluorescence standards for optically dilute solutions (A<0.05). Theoretical calculations were performed by Gaussian 16 program and 09 program[1] at B3LYP/6-311 G(d,p) level. Circular dichroism was measured on a JASCO J-810 spectrophotometer. Circularly polarized luminescence was measured on a JASCO CPL-300 spectrophotometer.

S2. Synthesis and characterization

Perylene bisanhydride **1**[2] and BINOL bisanhydride **4**[3] are known compounds and were synthesized according to literature methods.

Synthesis of ***tert*-butyl 4-(aminomethyl)benzylcarbamate (2)**:[4] *p*-xylylenediamine (500.0 mg, 3.67 mmol) and triethylamine (TEA, 0.5 mL) were added to a 250 mL round-bottom flask with 150 mL chloroform, then $(Boc)_2O$ (800.0 mg, 3.67 mmol) was dropwise added to the above solution at RT. The reaction mixture was stirred overnight. Afterwards, the solvent was removed under reduced pressure, and the crude product was purified by dry load column chromatography (silica gel, eluent CH_2Cl_2, and then CH_2Cl_2/MeOH = 20:1, v/v), a colorless solid was obtained. Yield: 35 % (303.5 mg, 1.30 mmol). ^{1}H-NMR (400 MHz, 298 K, d6-DMSO/$CDCl_3$ = 1:1, v/v): δ = 7.24 (d, *J* = 8.04 Hz, 2 H), 7.17 (d, *J* = 8.00 Hz, 2 H), 6.91 (t, *J* = 5.80 Hz, 1 H), 4.12 (d, *J* = 6.20 Hz, 2 H), 3.77 (s, 2 H), 1.37 (s, 9 H).

Synthesis of **perylene diamine 3**: Perylene bisanhydride **1** (100.0 mg, 0.10 mmol), *tert*-butyl 4-(aminomethyl)benzylcarbamate **2** (55.0 mg, 0.22 mmol) and imidazole (300.0 mg, 4.40 mmol) were added to 100 mL toluene in a 250 mL round-bottom flask. The mixture was refluxed under a nitrogen atmosphere for 16 h. After completion of the reaction, the mixture was cooled to RT and solvent was removed under reduced pressure. The crude product was washed with chloroform (100 mL) and 1 M HCl (100 mL). The organic phase was collected and dried over anhydrous Na_2SO_4. After filtration and removing the solvent under vacuum, the crude product was purified by column chromatography (silica gel, eluent CH_2Cl_2/MeOH = 20:1, v/v), giving a red solid (135.0 mg, 0.095 mmol). This perylene bismide compound (50.0 mg, 0.035 mmol) was further dissolved in 20 mL CH_2Cl_2 in a 100 mL round-bottom flask, then 3 mL trifluoroacetic acid (TFA) was added. The color of the solution changed from red to dark blue immediately. After stirring at RT for 30 min, the solvent was removed under reduced pressure, giving a dark red solid in a quantitative yield (50.7 mg, 0.035 mmol). ^{1}H-NMR (400 MHz, 298 K, d_6-DMSO): δ = 8.12 (s, 6 H), 7.92 (s, 4 H), 7.36 (d, *J* = 4.0 Hz, 8 H), 7.30 (d, *J* = 8.8 Hz, 8 H), 6.87 (d, *J* = 8.8 Hz, 8 H), 5.17 (s, 4 H), 4.00 (m, 4 H), 1.24 (s, 36H). ^{13}C-NMR (101 MHz, 298 K, d_6-DMSO): δ = 162.6, 158.2, 157.8, 155.2, 152.4, 147.0, 137.3, 132.8, 132.3, 128.9, 127.4, 126.7, 122.4, 119.4, 119.1, 119.0, 118.6, 41.9, 34.1, 31.1. ESI-MS (positive mode, acetonitrile/chloroform = 1:1, v/v): calculated for $C_{80}H_{76}N_4O_8Na$, m/z 1243.5561, found: $[M+Na]^+$, 1243.5531. Melting point: 266 °C.

Synthesis of **BBI-PBI cyclophane**: Perylene diamine **3** (84.0 mg, 0.058 mmol), racemic binaphthol bisanhydride **4** (26.0 mg, 0.058 mmol) and imidazole (1.1 g) were dispersed in 600 mL toluene in a 1000 mL round-bottom flask under a nitrogen atmosphere and the mixture was refluxed overnight. After cooling to RT, toluene was removed under vacuum, the residue was dissolved in chloroform (100 mL) and washed with 1 M HCl (100 mL). The organic phase was collected and dried over anhydrous Na_2SO_4. After filtration, the solvent was removed under vacuum and the product was purified by column chromatography (silica gel, eluent CH_2Cl_2/MeOH = 50:1) and recycling GPC to give a dark red solid, yield: 8 % (7.6 mg, 4.6 μmol). ^{1}H-NMR (600 MHz, 347.8 K, $C_2D_2Cl_4$): δ = 8.35 (s, 2 H), 8.28 (m, 2 H), 8.14 (d, *J* = 7.8 Hz, 4 H), 7.41 (m, 10 H), 7.29 (m, 10 H), 6.82 (br, 4 H), 6.72 (br, 4H), 5.44 (br, 2H), 5.34 (br, 6H), 3.82 (s, 6H), 1.35 (s, 18H), 1.33 (s, 18H). ^{13}C-NMR (150 MHz, 347.8 K, $C_2D_2Cl_4$): δ = 163.8, 163.7, 163.3, 163.2, 156.2, 153.2, 152.9, 147.7, 147.7, 137.4, 137.0, 132.7, 131.8, 131.5, 130.0, 129.8, 129.3, 129.3, 128.5, 127.7, 126.8, 126.8, 125.6, 124.8, 124.4, 123.9, 123.9, 123.2, 122.6, 122.5, 121.1, 120.7, 120.6, 120.5, 119.5, 119.2, 117.9, 116.9, 116.5, 99.9, 80.1, 80.0, 79.8, 57.1, 43.4, 43.1, 34.5, 34.5, 31.7, 31.7. HR-MALDI-TOF (pos. mode, chloroform): calculated for $[M+H]^+$, $C_{106}H_{87}N_4O_{14}$: m/z 1639.614, found: 1639.610. Melting point: 331 °C.

S3. Supplementary figures

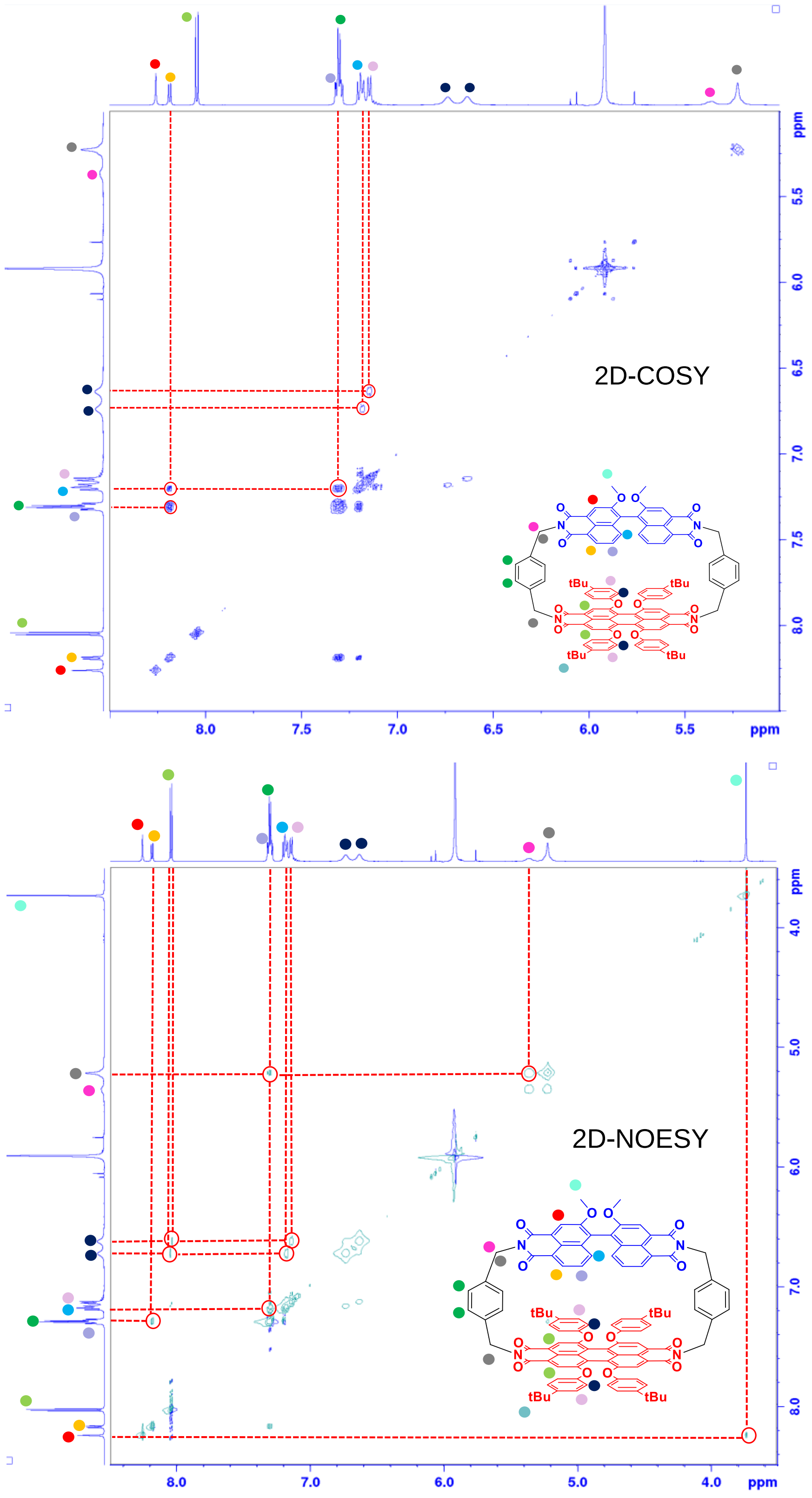

Figure S1. 2D-COSY and 2D-NOESY spectra of **BBI-PBI** cyclophane, [**BBI-PBI**] = 3.0 mM in $C_2D_2Cl_4$, 347.8 K, 600 MHz.

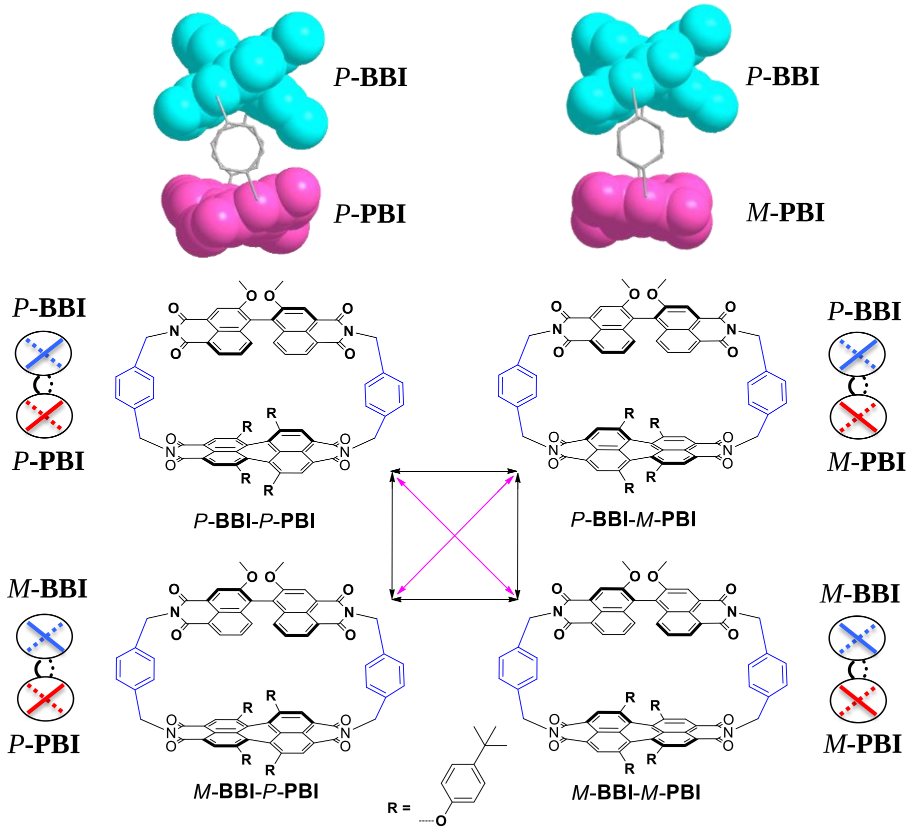

Figure S2. Chemical structures with enantiomeric and diastereomeric relationships for the **BBI-PBI** cyclophane stereoisomers. Magenta double arrows represent enantiomers. Dark double arrows represent diastereomers.

For better visualization, the side view CPK modes of *P*-**BBI**-*P*-**PBI** and *P*-**BBI**-*M*-**PBI** are shown at the top of each chemical structure. BBI is colored in cyan and PBI is colored in magenta. The 4-*tert*-butylphenoxy groups on bay position were omitted for clarity. Further, the cartoon figures on left and right sides represent the core twisting of BBI and PBI chromophores from a side view, in which solid and dashed blue lines indicate naphthalene imide planes of BBI, solid and dashed red lines indicate naphthalene imide planes of PBI.

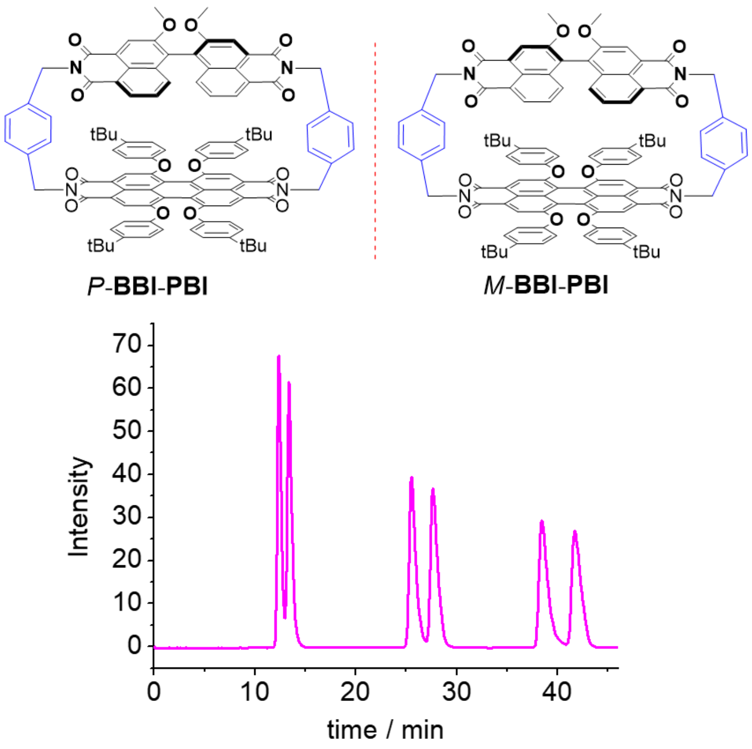

Figure S3. HPLC chromatogram for **BBI-PBI** cyclophane on a recycling semi-preparative Trentec Reprosil 100 chiral-NR column. Dichloromethane/hexane as eluent, v/v = 4:1, flow rate: 1 mL/min.

Both the binaphthol bisanhydride **4** and the tetraphenoxy-substituted PBI diamine **3** (Scheme 1 in main text) utilized in the synthesis are racemic, therefore, **BBI-PBI** cyclophane should consist of four isomers theoretically, including enantiomers and diastereomers, i.e. *P*-**BBI**-*P*-**PBI**, *P*-**BBI**-*M*-**PBI**, *M*-**BBI**-*P*-**PBI** and *M*-**BBI**-*M*-**PBI** (Fig. S2). However, the racemization barrier for tetraphenoxy-substituted PBI is only about 60 kJ/mol,[5] which is not high enough for enantiomeric resolution on a chiral HPLC column. As a result, only two compounds, *P*-**BBI-PBI** and *M*-**BBI-PBI** cyclophanes were obtained after resolution on a semi-preparative chiral column (Fig. S3), for which the *P*/*M*-ratio of the perylene subunit depends on the degree of chirality transfer imparted by the BBI unit.[3]

Table S1. Optical properties of reference compounds in CH_2Cl_2 at 293 K.

	λ_{abs}/nm	ε_{max}/M^{-1} cm^{-1}	λ_{em} / nm	$\Delta\tilde{\nu}_{Stokes}$ / cm^{-1}	FLQY[a]	Lifetime
ref-**BBI**	397	2.7 x 10^4	444	2666	0.68±0.05	3.6 ns[b]
ref-**PBI**	582	4.7 x 10^4	614	895	0.91±0.05	6.6 ns[c]

a. Perylene (FLQY 0.94 in cyclohexane) as a reference for ref-**BBI**, *N*,*N*′-bis(2,6-diisopropylphenyl)-1,6,7,12-tetraphenoxyperylene-3,4:9,10-bis(dicarboximide) (FLQY 0.96 in chloroform) for ref-**PBI**. **b**. Diode laser excitation wavelength 378.2 nm, monitored emission wavelength for decay at 444 nm. **c**. Diode laser excitation wavelength 505.8 nm, monitored emission wavelength for decay at 614 nm.

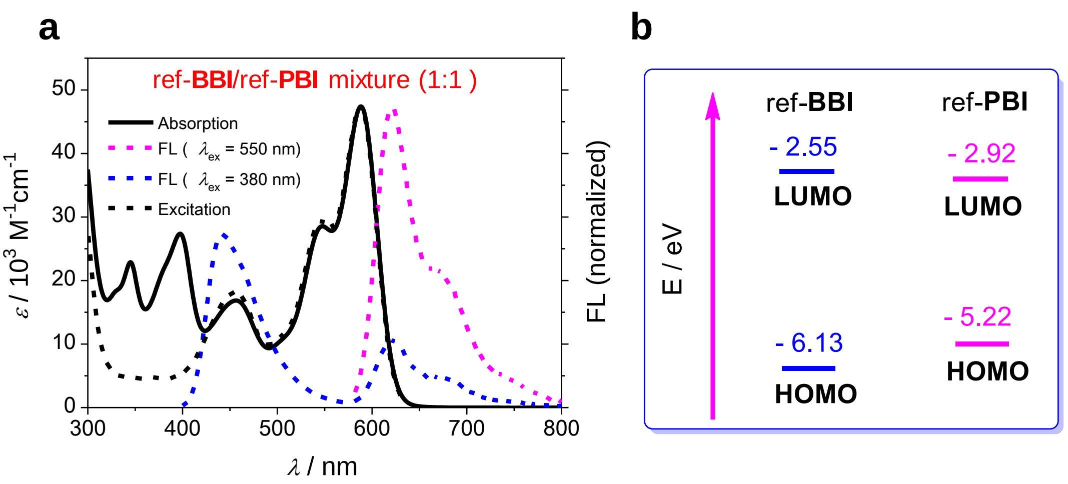

Figure S4. a) Optical spectra of the mixture of ref-**BBI** and ref-**PBI** (1:1 in molar ratio) in CH_2Cl_2 at 293K. For UV-Vis measurements, [ref-**BBI**] = [ref-**PBI**] = 2.4 × 10^{-5} M. The fluorescence spectra were obtained by photoexcitation at 380 nm (blue dotted line) and 550 nm (magenta dotted line), respectively. The excitation spectrum (black dashed line) was measured for the emission wavelength at 650 nm. b) HOMO and LUMO energy levels of reference compounds calculated by DFT, Gaussian 16, at B3LYP/6-311G(d,p) level.

In addition to the optical properties of the two individual dyes, also those for the ref-**BBI**/ref-**PBI** mixture (1:1 in molar ratio) were recorded. Here upon excitation of the PBI chromophore at 550 nm, only the PBI emission band was observed (Fig. S4a, magenta dotted line) whilst upon excitation at 380 nm mainly BBI emission was observed with relatively weak PBI emission (Fig. S4a, blue dotted line). The latter originates from direct photoexcitation of the weak absorption band of ref-**PBI** at 330-400 nm. These results indicate that the absence of energy transfer from ref-**BBI** to ref-**PBI** in dilute solution. This conclusion is further supported by the excitation spectra (Fig. S4a, black dashed line), which show that the absorption of ref-**BBI** at 330-400 nm is not responsible for PBI emission.

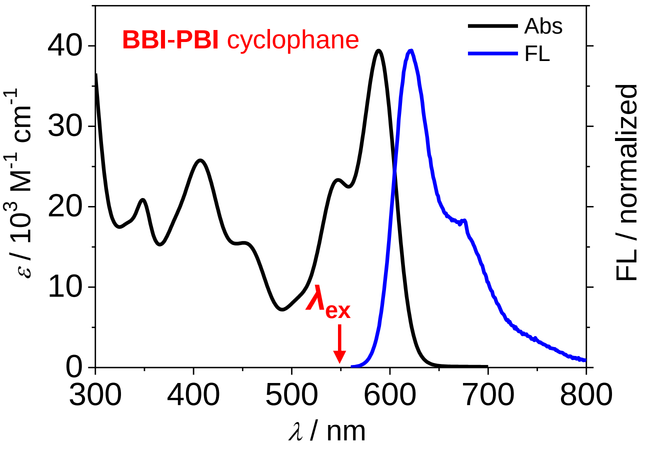

Figure S5. UV-Vis and FL spectra (λ_{ex} = 550 nm) of *P*-**BBI-PBI** at a concentration of 1.8×10^{-5} M in CH_2Cl_2 (for UV-Vis), 293 K.

When directly exciting the acceptor PBI at 550 nm, the emission curve of BBI-PBI cyclophane (Fig. S5, blue line) are almost identical to that of individual ref-**PBI** chromophore (Fig. 2a in main text, blue dashed line).

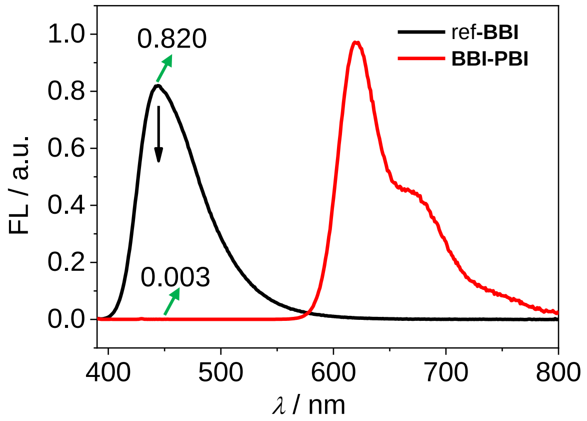

Figure S6. Emission spectra of ref-**BBI** (dark line) was normalized with *P*-**BBI-PBI** cyclophane (red line), λ_{ex} = 380 nm. [ref-**BBI**] = [*P*-**BBI-PBI**] = 1.0×10^{-6} M in CH_2Cl_2, 293 K.

FRET efficiency (Φ_T) is evaluated by the following equation:

$\Phi_T = 1 - I_{DA}/I_D$, where I_{DA} and I_D represent the fluorescence intensity of BBI in the presence and absence of PBI.

We obtain Φ_T = 1 - 0.003/0.820 = 99.6 %.

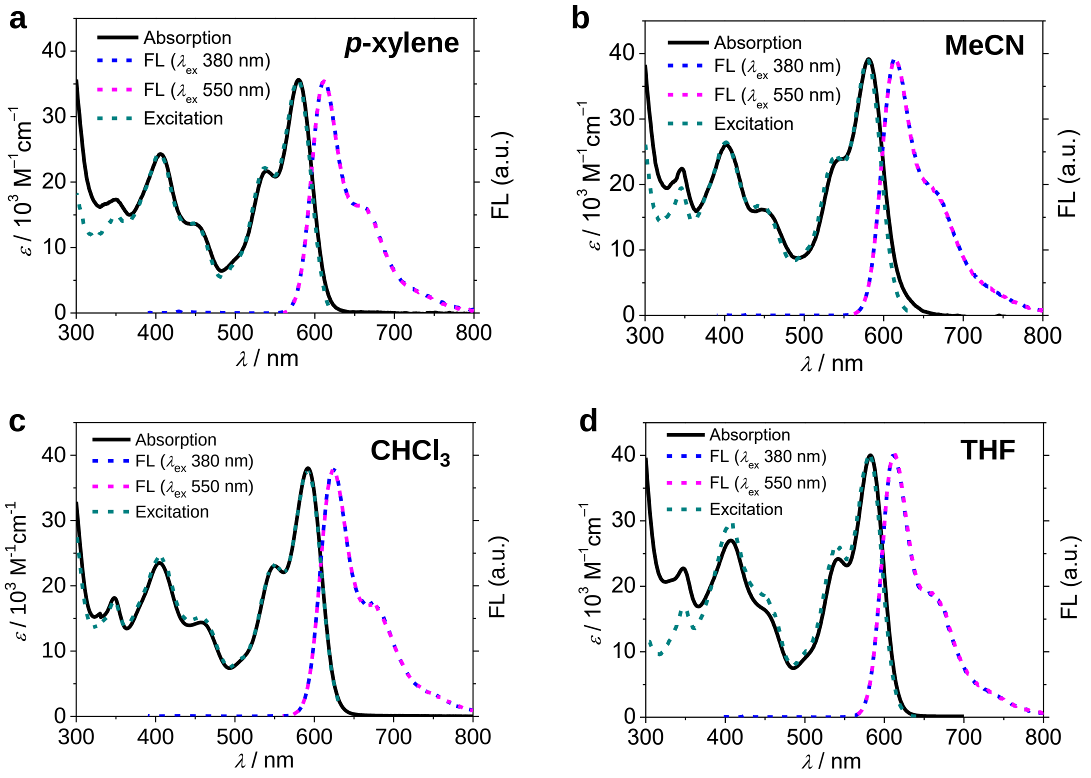

Figure S7. UV-Vis, FL and excitation spectra of *P*-**BBI-PBI** cyclophane in different solvents, a) *p*-xylene, b) MeCN, c) $CHCl_3$, and d) THF. [*P*-**BBI-PBI**] = 1.8×10^{-5} M for UV-Vis, 293 K. Black solid lines, absorption spectra. Green dashed lines, excitation spectra (emission wavelength at 650 nm). Magenta dashed lines, emission spectra (λ_{ex} = 550 nm). Blue dashed lines, emission spectra (λ_{ex} = 380 nm). Emission spectra are normalized according to corresponding absorption maxima.

Table S2. Optical properties of *P*-**BBI-PBI** in different solvents.

	λ_{abs} / nm	$\varepsilon_{max}/10^4$ $M^{-1}cm^{-1}$	λ_{em} / nm	$\Delta\tilde{\nu}_{Stokes}$ / cm^{-1}	**Lifetime**[a] PBI excitation	**Lifetime**[b] BBI excitation	**FLQY**[c] PBI excitation /± 0.05	**FLQY**[d] BBI excitation /± 0.05
$CHCl_3$	592	3.8	624	866	7.4 ns	7.4 ns	0.98	0.83
p-xylene	579	3.5	611	905	6.9 ns	6.9 ns	0.80	0.87
CH_2Cl_2	589	3.9	621	875	7.6 ns	7.6 ns	0.87	0.92
THF	582	4.0	614	748	7.7 ns	7.7 ns	0.92	0.98
MeCN	581	3.9	614	925	7.5 ns	7.5 ns	0.83	0.93

a. Diode excitation wavelength 505.8 nm, monitored PBI emission wavelength 650 nm; **b**. Diode excitation wavelength 378.2 nm, monitored PBI emission wavelength 650 nm; **c**. *N*,*N*'-bis(2,6-diisopropylphenyl)-1,6,7,12-tetraphenoxy perylene -3,4:9,10-bis(dicarboximide) ("Perylene Red", QY 0.96 in chloroform) used as reference **d**. Coumarin 153 (QY 0.90 in cyclohexane) used as a reference.

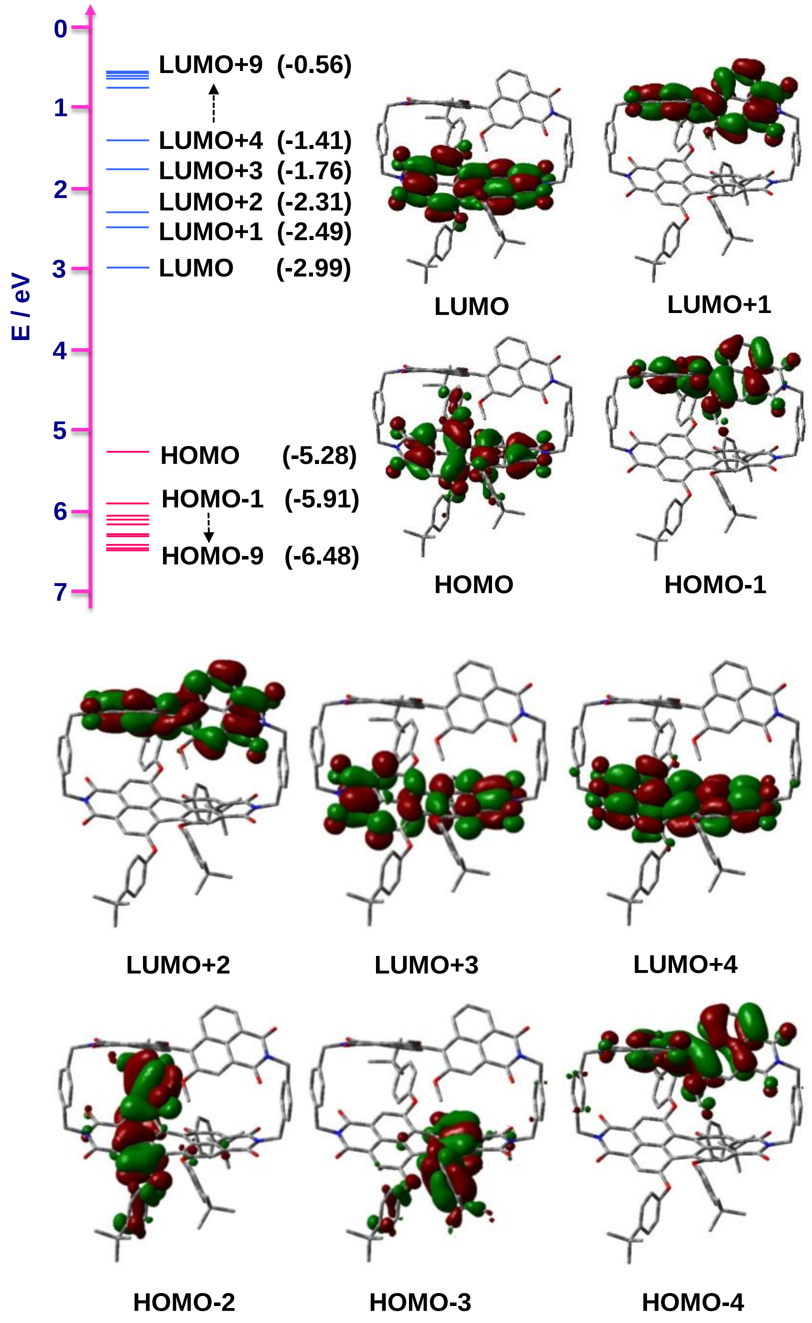

Figure S8. Energy levels and molecular orbitals of *P*-**BBI**-*M*-**PBI** cyclophane calculated by Gaussian 16 at B3LYP 6-311G (d,p) level.

Table S3. Molecular orbital (MO) transition contribution to excited states of *P*-**BBI**-*M*-**PBI** cyclophane, calculated by TD-DFT, Gaussian 09, B3LYP/6-311 G(d,p).

States	MO contribution	Energy	Wavelength	Oscillator strength
S1	HOMO → LUMO (99.2%)	2.07 eV	599 nm	0.391
S2	HOMO → LUMO+1 (99.2%)	2.45 eV	506 nm	< 0.001
S3	HOMO-1 → LUMO (99.6%)	2.56 eV	484 nm	< 0.001
S10	HOMO-1 → LUMO+1 (93.9%)	2.91 eV	425 nm	0.185

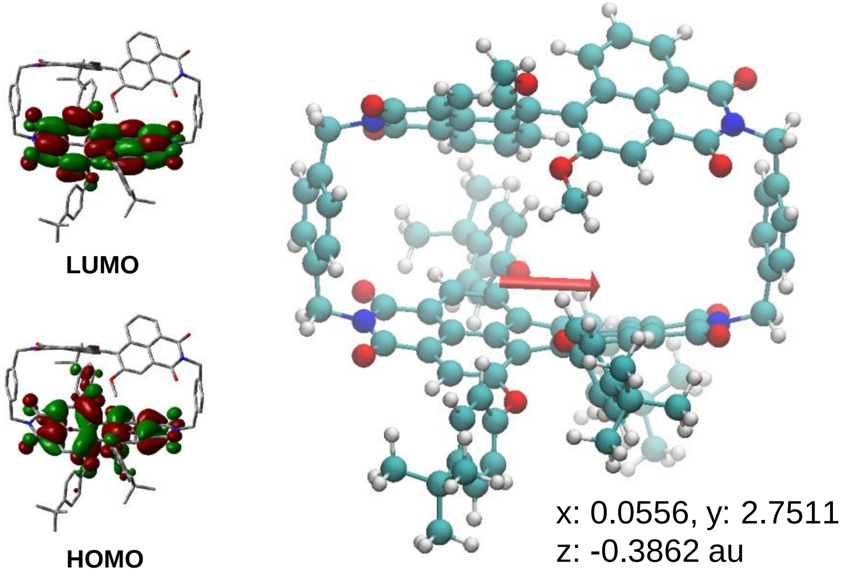

Figure S9. Calculated transition dipole moment (red arrow)[6,7] for the HOMO-LUMO transition of *P*-**BBI**-*M*-**PBI** cyclophane, TD-DFT, Gaussian 09 at B3LYP 6-311 G(d,p) level.

TD-DFT calculations indicate that the major MO contribution to the S1 state is the HOMO-LUMO transition (Table S3). These two frontier orbitals are mainly located on the PBI chromophore (Fig. S9). Accordingly, the transition dipole moment of the S_0-S_1 transition of *P*-**BBI**-*M*-**PBI** cyclophane is polarized along the long axis of PBI, which is only slightly interfered by the presence of the BBI chromophore.

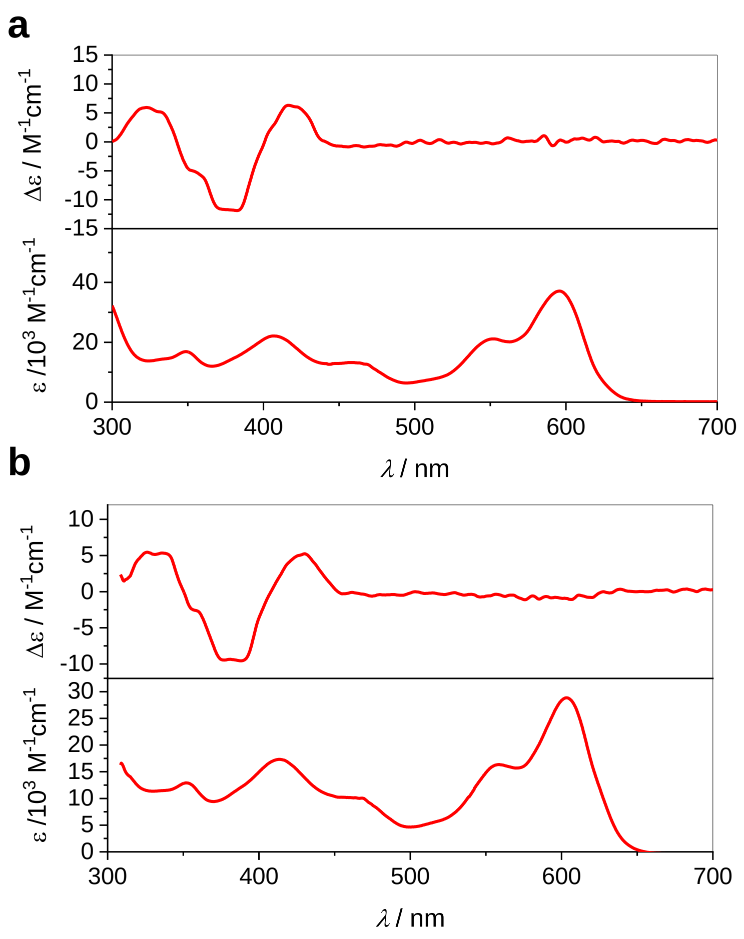

Figure S10. CD spectra of *P*-**BBI-PBI** cyclophane in a) $CHCl_3$ (stabilized with amylene, filtered over Alox), and b) $CHBr_3$ (stabilized with 4 wt% EtOH), [*P*-**BBI-PBI**] = 2.6×10^{-4} M, cuvette path length 1 mm, 293K. The results are similar to those in $CHCl_3$ stabilized with 4 wt% ethanol (shown in Fig. 3a in main text).

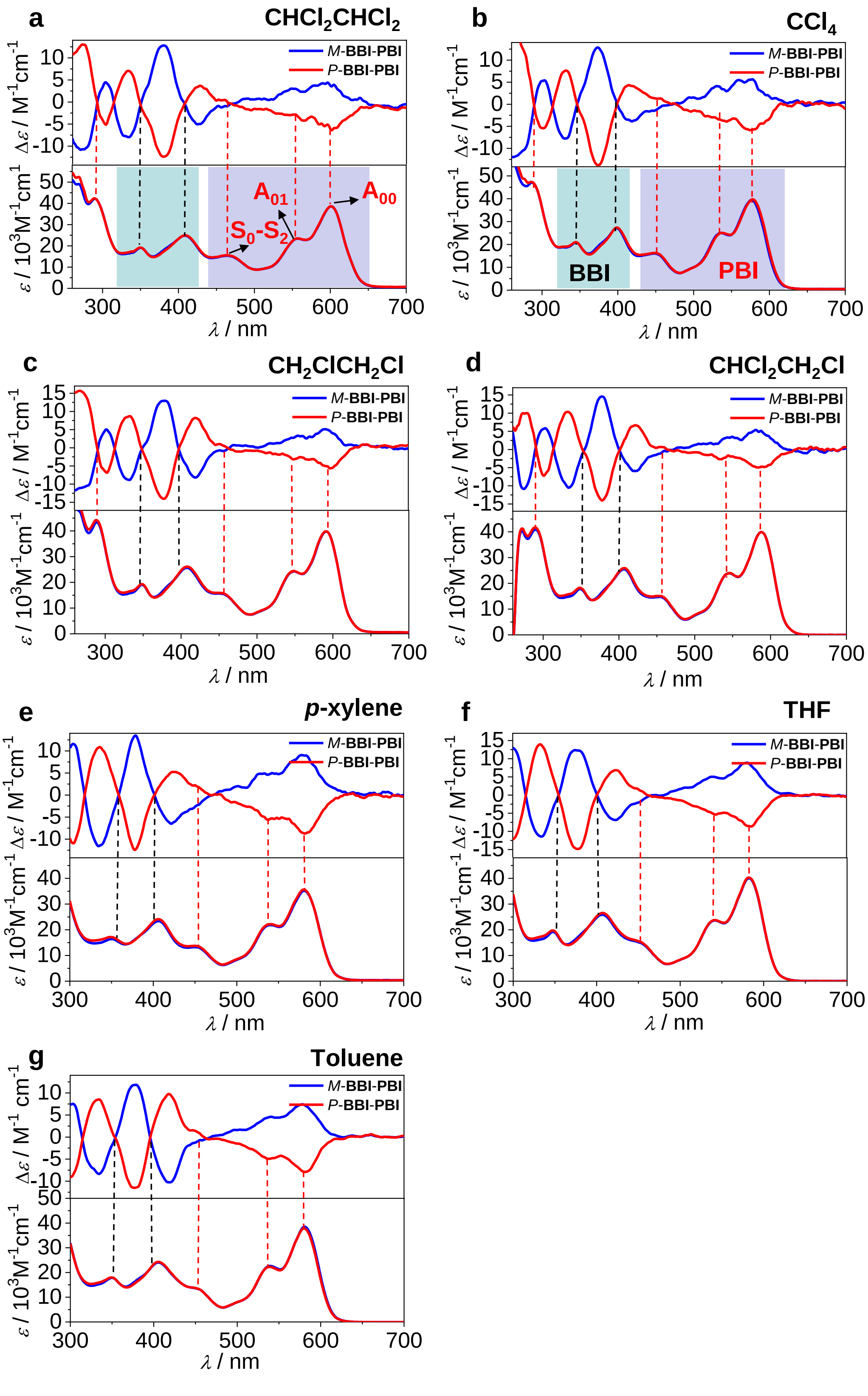

Figure S11. CD and UV/Vis spectra of **BBI-PBI** cyclophane in various solvents. [**BBI-PBI**] = 2.6 × 10^{-4} M, cuvette path length 1 mm, 293 K.

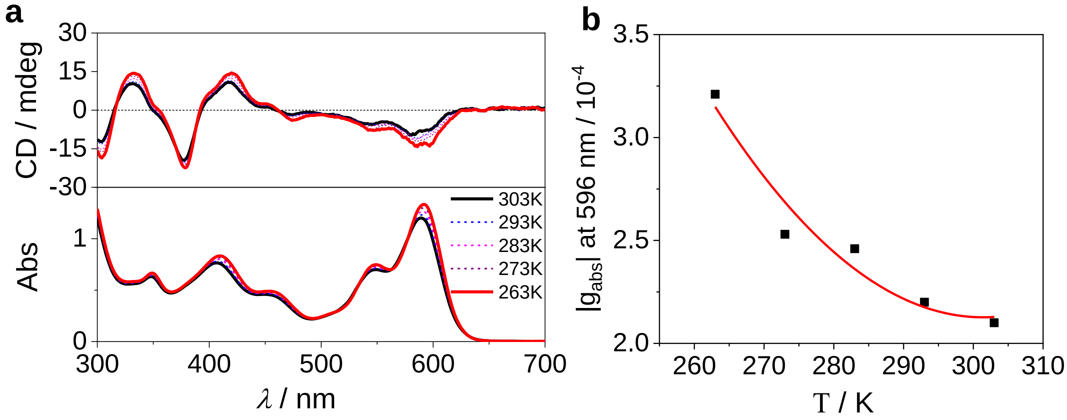

Figure S12. a) Variable-temperature CD spectra of *P*-BBI-PBI cyclophane in CH_2Cl_2. Concentration at 293 K is 3.16×10^{-4} M, cuvette path length is 1 mm. b) Plot of absorption dissymmetry factor ($|g_{abs}|$) of the cyclophane at 596 nm against temperature. $|g_{abs}|$ is calculated by the following equation: $|g_{abs}|$ = CD/(32980×Abs.), where CD and Abs. values are obtained from Fig. S12a.

The ee value of PBI subcomponent at 263 K is estimated to be $3.24\times10^{-4}/0.0018 = 0.18$, where 0.0018 is the absorption dissymmetry factor of an enantiopure *P*-helical tetraphenoxy-substituted PBI based on our previous report.[5]

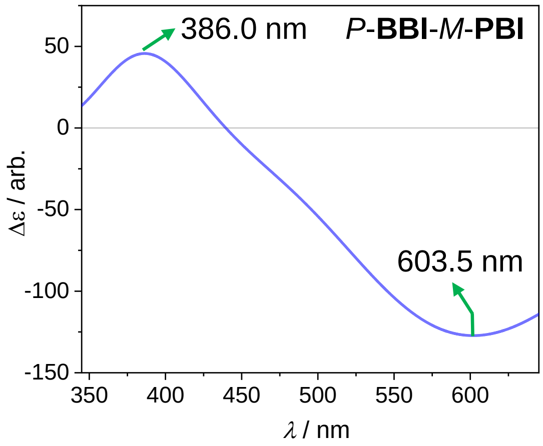

Figure S13. Calculated CD spectrum of *P*-**BBI**-*M*-**PBI** cyclophane without consideration of the solvent, TD-DFT, Gaussian 09, B3LYP/6-311+G(d,p) level.

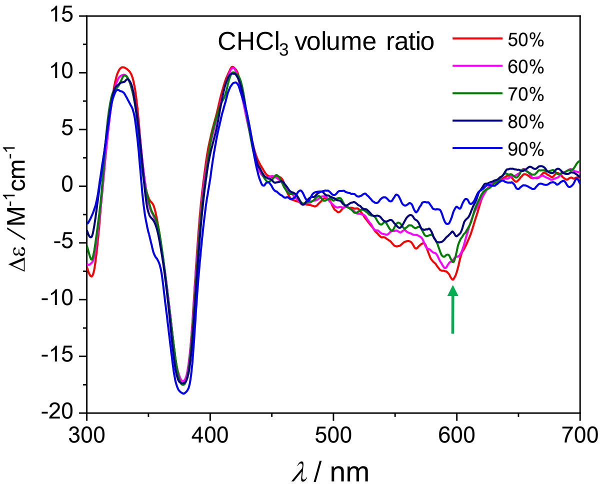

Figure S14. CD spectra of *P*-**BBI-PBI** in $CHCl_3/CH_2Cl_2$ co-solvents with different $CHCl_3$ volume ratios. [*P*-**BBI-PBI**] = 1.7 × 10^{-5} M, cuvette path length 10 mm, 293 K.

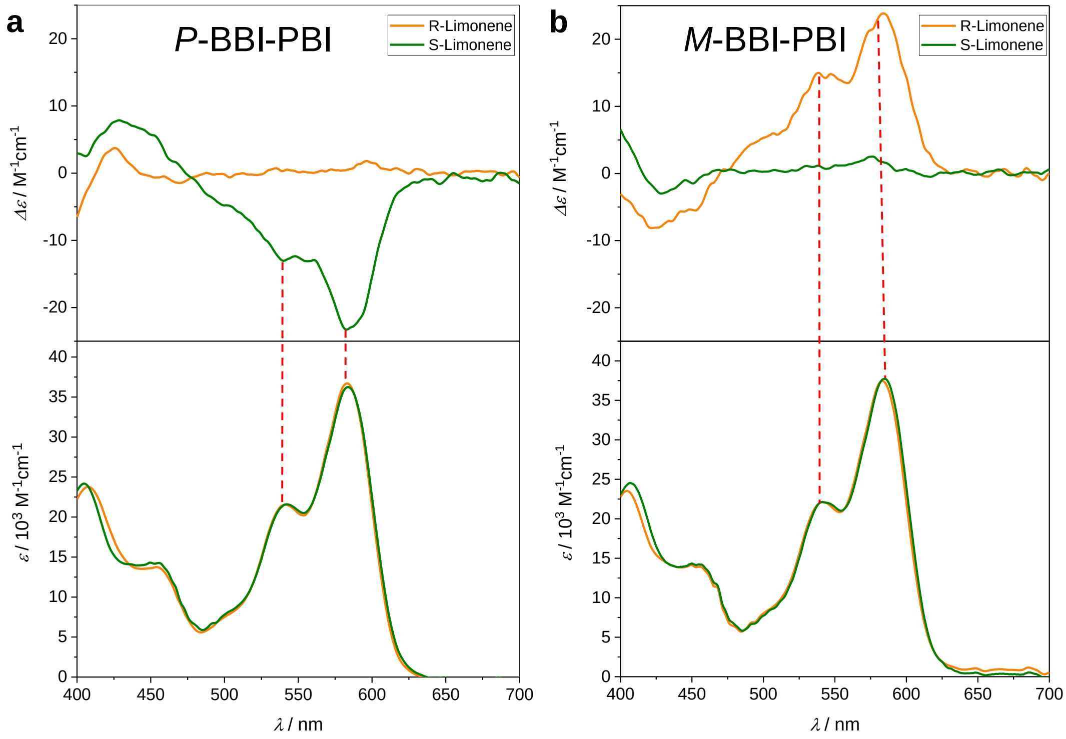

Figure S15. CD spectra of a) *P*-**BBI-PBI** and b) *M*-**BBI-PBI** cyclophanes in *R*-limonene and *S*-limonene. [**BBI-PBI**] = 2.5 × 10^{-5} M, cuvette path length 10 mm, 293 K.

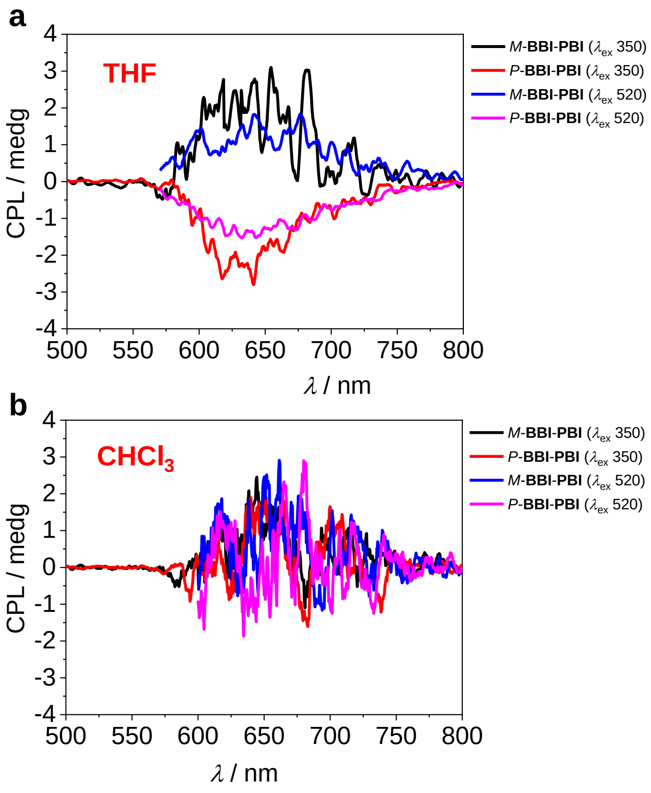

Figure S16. CPL spectra of *P*-**BBI-PBI** and *M*-**BBI-PBI** isomers in a) THF, and b) $CHCl_3$. [**BBI-PBI**] = 2.3×10^{-4} M, cuvette path length 1 mm, 293 K. Black and red lines are obtained by photoexcitation at 350 nm, blue and magenta lines are obtained by photoexcitation at 520 nm.

S4. Supplementary spectra

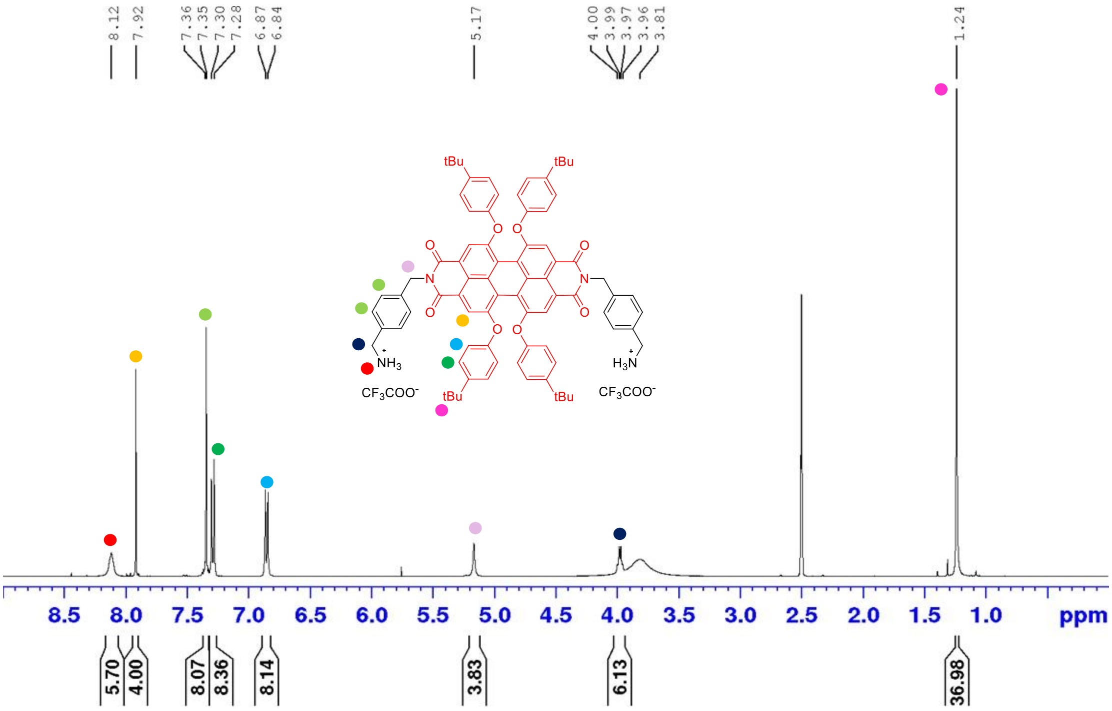

Figure S17. ^{1}H-NMR (400 MHz, 298 K, d_6-DMSO) spectrum of **perylene diamine 3**.

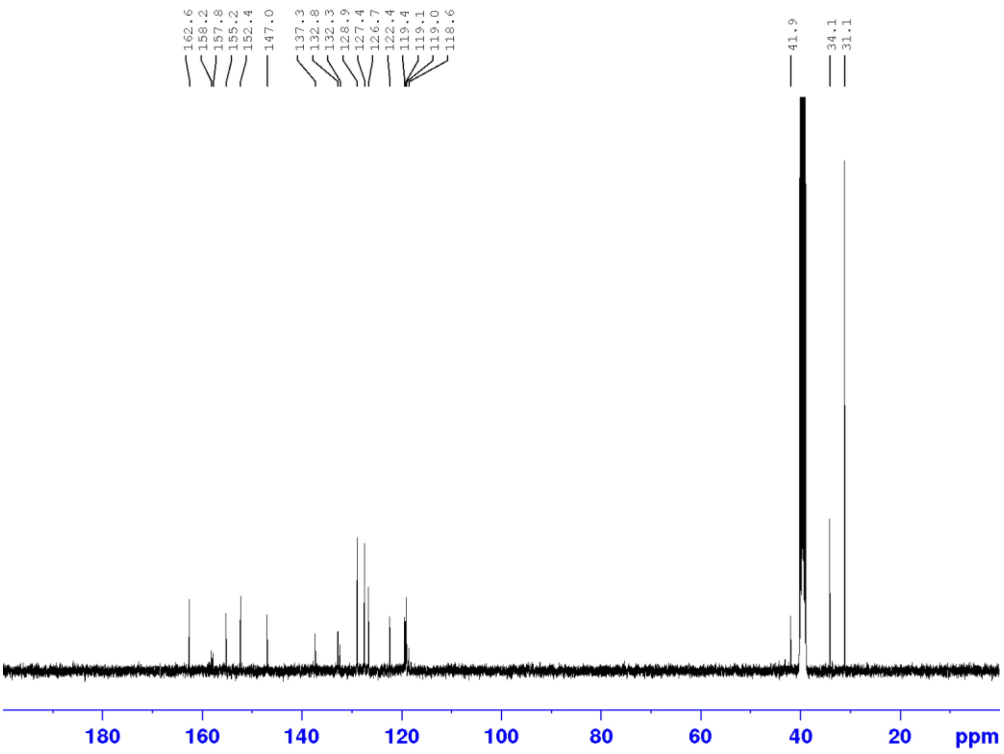

Figure S18. ^{13}C-NMR (101 MHz, 298 K, d_6-DMSO) spectrum of **perylene diamine 3**.

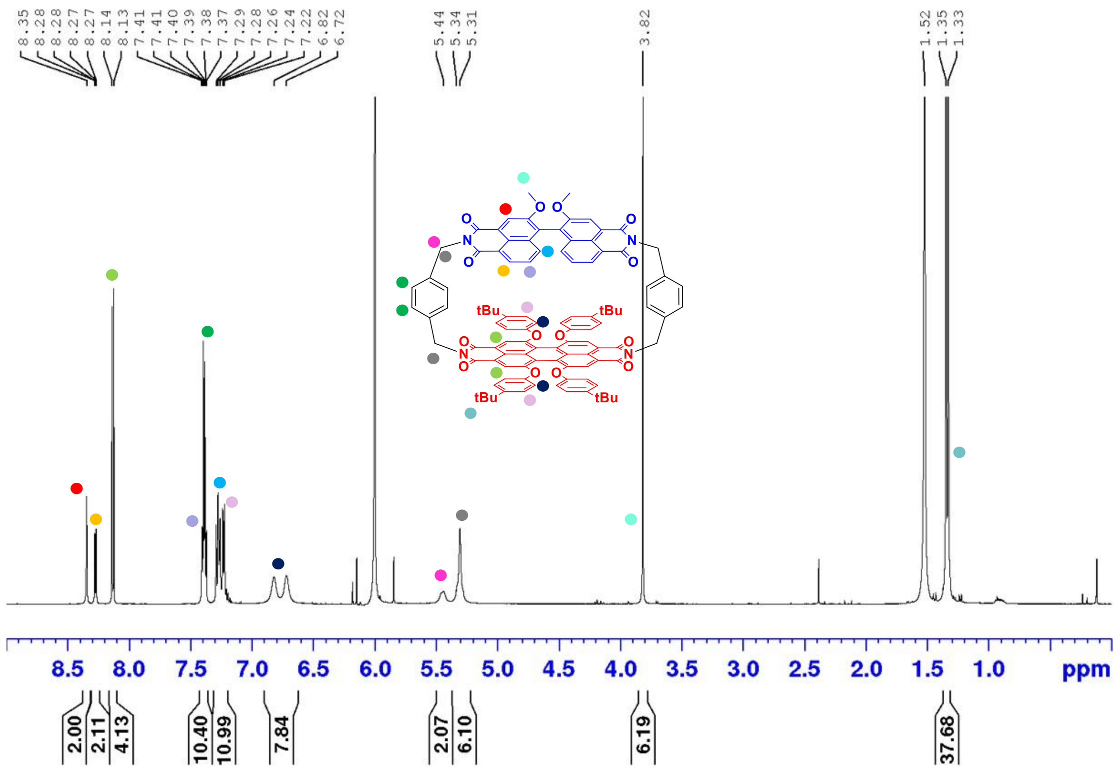

Figure S19. ^{1}H-NMR (600 MHz, 347.8 K, $C_2D_2Cl_4$) spectrum of **BBI-PBI** cyclophane.

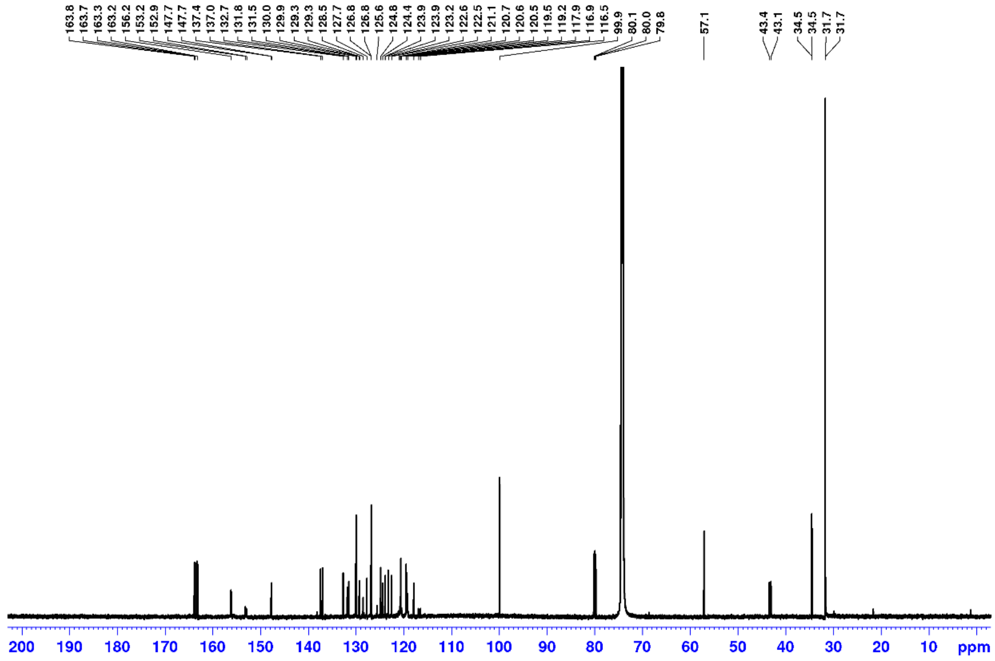

Figure S20. ^{13}C-NMR (150 MHz, 347.8 K, $C_2D_2Cl_4$) spectrum of **BBI-PBI** cyclophane.

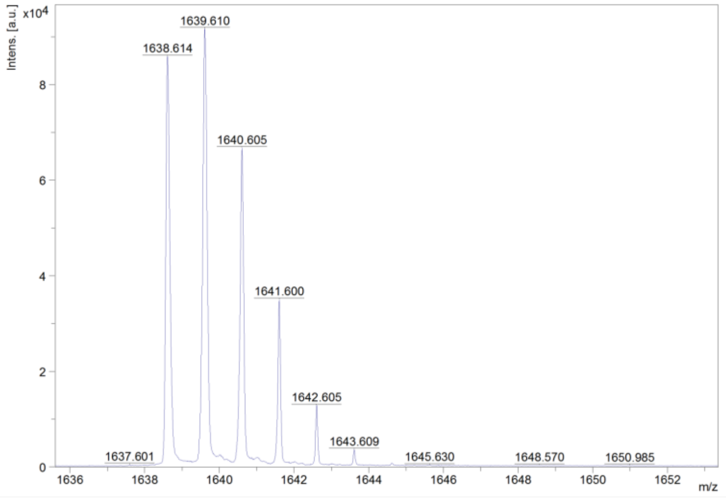

Figure S21. HR-MALDI-TOF (pos. mode, chloroform) spectrum of **BBI-PBI** cyclophane.

S5. References

1. M. J. Frisch, G. W. Trucks, H. B. Schlegel, G. E. Scuseria, M. A. Robb, J. R. Cheeseman, G. Scalmani, V. Barone, G. A. Petersson, H. Nakatsuji, X. Li, M. Caricato, A. V. Marenich, J. Bloino, B. G. Janesko, R. Gomperts, B. Mennucci, H. P. Hratchian, J. V. Ortiz, A. F. Izmaylov, J. L. Sonnenberg, Williams, F. Ding, F. Lipparini, F. Egidi, J. Goings, B. Peng, A. Petrone, T. Henderson, D. Ranasinghe, V. G. Zakrzewski, J. Gao, N. Rega, G. Zheng, W. Liang, M. Hada, M. Ehara, K. Toyota, R. Fukuda, J. Hasegawa, M. Ishida, T. Nakajima, Y. Honda, O. Kitao, H. Nakai, T. Vreven, K. Throssell, J. A. Montgomery Jr., J. E. Peralta, F. Ogliaro, M. J. Bearpark, J. J. Heyd, E. N. Brothers, K. N. Kudin, V. N. Staroverov, T. A. Keith, R. Kobayashi, J. Normand, K. Raghavachari, A. P. Rendell, J. C. Burant, S. S. Iyengar, J. Tomasi, M. Cossi, J. M. Millam, M. Klene, C. Adamo, R. Cammi, J. W. Ochterski, R. L. Martin, K. Morokuma, O. Farkas, J. B. Foresman, D. J. Fox, Gaussian 16 Revision C.01 and Gaussian 09 Revision D.01.
2. D. Dotcheva, M. Klapper, K. Müllen, *Macromol. Chem. Phys*. **1994**, *195*, 1905-1911.
3. M. Chen, Y. Zhang, M. Vysotsky, J. Lindner, M. Li, M. J. Lin, F. Würthner, *Org. Chem. Front*. **2019**, *6*, 3731-3740.
4. T. Naranjo, K. M. Lemishko, S. de Lorenzo, A. Somoza, F. Ritort, E. M. Perez, B. Ibarra, *Nat. Commun*. **2018**, *9*, 4512.
5. P. Osswald, M. Reichert, G. Bringmann, F. Würthner, *J. Org. Chem*. **2007**, *72*, 3403-3411.
6. T. Lu, F. Chen, *J. Comput. Chem*. **2012**, *33*, 580-592.
7. W. Humphrey, A. Dalke, K. Schulten, *J. Molec. Graphics*, **1996**, *14*, 33-38.
8. N. Harada, K. Nakanishi, Circular Dichroic Spectroscopy: Exciton Coupling in Organic Stereochemistry. *University Science Books*, Mill Valley, CA, **1983**.
